# Changes in Surgical Site Infections after Living Donor Liver Transplantation

**DOI:** 10.1371/journal.pone.0136559

**Published:** 2015-08-31

**Authors:** Masaki Yamamoto, Shunji Takakura, Yoshitsugu Iinuma, Go Hotta, Yasufumi Matsumura, Aki Matsushima, Miki Nagao, Kohei Ogawa, Yasuhiro Fujimoto, Akira Mori, Yasuhiro Ogura, Toshimi Kaido, Shinji Uemoto, Satoshi Ichiyama

**Affiliations:** 1 Department of Clinical Laboratory Medicine, Kyoto University Graduate School of Medicine, Kyoto, Japan; 2 Department of Infectious Diseases, Kanazawa Medical University, Kanazawa, Japan; 3 Division of Hepato-Biliary-Pancreatic Surgery and Transplantation, Department of Surgery, Kyoto University Graduate School of Medicine, Kyoto, Japan; 4 Department of Transplantation Surgery, Nagoya University Hospital, Nagoya, Japan; University of Colorado, UNITED STATES

## Abstract

Surgical site infections (SSIs) are a major threat for liver transplant recipients. We prospectively studied SSIs after living donor liver transplantation (LDLT) at Kyoto University Hospital from April 2001 to March 2002 (1^st^ period) and from January 2011 to June 2012 (2^nd^ period). We investigated the epidemiology of SSIs after LDLT and determined the differences between the two periods. A total of 129 adult recipients (66 during the 1^st^ period and 63 during the 2^nd^ period) and 72 pediatric recipients (39 and 33) were included in this study. The SSI rates for each period were 30.3% (1^st^ period) and 41.3% (2^nd^ period) among the adult recipients and 25.6% and 30.3% among the pediatric recipients. The overall rates of 30-day mortality among adult transplant recipients with SSIs were 10.0% (1^st^ period) and 3.9% (2^nd^ period). No pediatric recipient died from SSIs after LDLT in either period. The incidence of *Enterococcus faecium* increased from 5.0% to 26.9% in the adults and from 10.0% to 40.0% in the pediatric patients. Extended-spectrum β-lactamase-producing Enterobacteriaceae were emerging important isolates during the 2^nd^ period. For this period, a univariate analysis showed that ABO incompatibility (*P* = 0.02), total operation duration (*P* = 0.01), graft-to-recipient body weight ratio (GRWR [*P* = 0.04]), and Roux-en-Y biliary reconstruction (*P*<0.01) in the adults and age (*P* = 0.01) and NHSN risk index (*P* = 0.02) in the children were associated with SSI development. In a multivariate analysis, lower GRWR (*P* = 0.02) and Roux-en-Y biliary reconstruction (*P*<0.01) in the adults and older age (*P* = 0.01) in the children were independent risk factors for SSIs during the 2^nd^ period. In conclusion, SSIs caused by antibiotic resistant bacteria may become a major concern. Lower GRWR and Roux-en-Y biliary reconstruction among adult LDLT recipients and older age among pediatric LDLT recipients increased the risk of developing SSIs after LDLT.

## Introduction

Living donor liver transplantation (LDLT) is a useful strategy for end-stage liver disease and was initially developed for pediatric liver transplantation [1]. This technique has been used in adult patients and has been primarily been developed in Asia because of an ongoing critical shortage of cadaveric grafts [2]. The major indications of LDLT include biliary atresia, other pediatric liver diseases, and various adult liver diseases (e.g., hepatitis virus-related liver disease with or without hepatocellular carcinoma [HCC], primary biliary cirrhosis, and primary sclerosing cholangitis). Infection is a major complication of liver transplantation because of the procedure’s technical complexity, long operation duration, and the potential for bacterial contamination from the gastrointestinal tract [3–6]. Surgical site infection (SSI) is one of the most common infectious complications of liver transplantation, and its incidence has been reported to be between 18% and 38% [7–9].

We previously conducted a prospective study at Kyoto University Hospital from April 2001 to March 2002 to determine the risk factors for SSIs after LDLTs performed [7]. In the 10 years since that study, there have been several changes in antimicrobial prophylaxis and immunosuppression protocols in LDLT. The previous study revealed the importance of *Enterococcus faecalis* as a causative pathogen of SSIs after LDLT. Therefore, in 2003, we changed the perioperative antibacterial prophylaxis from flomoxef, an oxacephem antibiotic agent available in Japan, to ampicillin and cefotaxime to target *E*. *faecali*s, as well as Enterobacteriaceae and methicillin-susceptible *Staphylococcus aureus*. In the 10 years since, a few studies have reported the epidemiology and risk factors of SSIs after LDLT, but sufficient data have yet not been accumulated [10]. The aims of this prospective study were to update the epidemiology of SSIs after LDLT and determine the differences between the two study periods.

## Materials and Methods

### Study Population and SSI Definition

We aimed to collect almost the same number of SSI cases at the start of the current study (2^nd^ period) that we included in our previous study (1^st^ period). The prospective study population included 129 adult recipients (66 during the 1^st^ period and 63 during the 2^nd^ period) and 72 pediatric recipients (39 and 33) at the Department of Transplantation Surgery, Kyoto University Hospital. Adults were defined as individuals aged 18 years or older. All recipients who received an LDLT were prospectively followed by 2 infection control physicians, and all infections from the time of surgery until 30 days after the LDLT were recorded. All of the data were predefined and collected using case report forms. We followed up within 30 days after the LDLT. Nine recipients (5 adult recipients and 4 pediatric recipients during the 1^st^ period) were discharged within 30 days, and all were followed up at the outpatient department. We included only recipients who had received a primary LDLT and included only the first episode of SSI in the analyses for this study. The Institutional Review Board of Kyoto University Hospital approved this study protocol. No informed consent needed because the data were analyzed anonymously.

SSI was defined in accordance with the Centers for Disease Control and Prevention (CDC) criteria as described in the previous study and with a criterion of onset within 30 days of surgical procedures (National Healthcare Safety Network [NHSN] definition) [7,11–13]. An abscess was defined as a collection of fluid, drained surgically or aspirated under ultrasound guidance, which showed pus cells upon miroscopy and for which culture yielded one or more organisms. Peritonitis was diagnosed if the ascitic fluid neutrophil count was greater than 250 cells/mm^3^ and if a pathogen was isolated. In all cases, intraabdominal abscesses were excluded using ultrasound scanning. Cholangitis was defined when there was one or more clinical indicators of infection (temperature >38°C or a white blood cell count >15×10^9^ /L) with an otherwise unexplained elevation of liver function tests concomitant with the repeated isolation of an organism in pure cultures from T-tube bile. These 3 types of infections were included in the space/organ criteria. We included the surgical incision site and the drain site infection as affected areas for SSI.

### Antimicrobial Prophylaxis and Immunosuppressive Treatments

Perioperative antibacterial prophylaxis consisted of flomoxef for 72 hr during the 1^st^ period and ampicillin and cefotaxime for 72 hr during the 2^nd^ period. This represented a major change in the antibacterial prophylaxis protocol between the two study periods. Trimethoprim and sulfamethoxazole were administered once daily as a prophylaxis against pneumocystis during immunosuppressant use. Fluconazole or micafungin was administered after transplantation as an antifungal prophylaxis at the surgeons’ discretion, and there was no difference in the antifungal prophylaxis used for each patient group and in each study period. The mean duration of antifungal prophylaxis was 24.8 days. The basic immunosuppression regimen consisted of tacrolimus and low-dose corticosteroid. Supplemental immunosuppression, when required, consisted of azathioprine, mizoribine, or mycophenolate mofetil with or without occasional induction therapy with monocronab-CD3 [7,14]. For ABO-incompatible recipients, a new immunosuppression protocol has been in use since 2004 [14]. This protocol consists of preoperative anti-CD 20 antibodies with preoperative plasma exchange to lower the anti-AB antigen titer, perioperative mycophenolate mofetil starting 7 days before the LDLT, and continuous postoperative intraportal administration of steroids until postoperative day 7.

### Clinical Characteristics and Risk Factors for SSIs

Demographic data, the potential risk factors for developing SSI, and outcomes were assessed. The following data were collected in the case reports: (i) pretransplant variables, including, gender, obesity [adult: body mass index (BMI) > 25; children and adolescents aged 2–19 years: BMI-for-age charts; and infants: weight-for-length charts], previous Roux-en-Y biliary reconstruction, previous use of renal replacement therapy, ABO incompatibility, serum albumin concentration, serum bilirubin concentration, pretransplantation intensive care unit stay (2^nd^ period), moderate or massive ascites, Child-Pugh score, and Model for End-Stage Liver Disease (MELD)/Pediatric End-Stage Liver Disease (PELD) score; and (ii) operative and posttransplant variables, including the duration of transplant surgery, intraoperative red blood cell transfusions, the graft-to-recipient body weight ratio (GRWR, 2^nd^ period), liver segment (2^nd^ period), type of biliary reconstruction, repeat intraabdominal or intrathoracic surgery, and the NHSN risk index. Using the NHSN risk index, each operation was scored from 0 to 3 based on the number of risk factors present in each recipient. These risk factors included having an American Society of Anesthesiologists’ physical status classification score of 3, 4, or 5; an operation classified as either contaminated or dirty/infected; and an operation that lasted longer than 13 hr, which was the 75^th^ percentile of the duration of 95 consecutive LDLTs performed at our hospital in 2000.

### Statistical Analysis

Statistical analyses were performed using STATA version 11.2 (StataCorp, College Station, TX, USA). Fisher’s exact or Pearson’s chi-square test was used to compare categorical variables as appropriate. Student’s t test was used to determine the statistical significance of continuous variables with a normal distribution, and the Mann-Whitney U test was used to test for the statistical significance of non-parametric continuous variables. The Kaplan–Meier method was used for the analysis of the SSI incidence in each study period, and the log-rank test was used to compare the difference. Cox proportional hazards models were used to analyze the risk factors for SSIs. The variables included in multivariate analyses were those that met the criterion of a *P* value <0.05 using forward variable selection. In the Cox models, SSIs after an LDLT were treated as time-dependent variables. The Gronnesby and Borgan test was performed to determine how well the final model reflected the data from which it was generated. A *P* value <0.05 was considered statistically significant.

## Results

### Characteristics of the Study Population

One hundred twenty-nine adult recipients (66 recipients during the 1^st^ period, and 63 recipients during the 2^nd^ period) and 72 pediatric recipients (39 recipients during the 1^st^ period, and 33 recipients during the 2^nd^ period) underwent primary LDLT during the two study periods. The demographic and clinical characteristics of these study patients are shown in Table 1.

**Table 1:** Demographic and clinical characteristics of the study patients.

Patient characteristics	Adult recipients				Pediatric recipients			
	2001–2002	2011–2012	Overall	*P* value	2001–2002	2011–2012	Overall	*P* value
	(n = 66)	(n = 63)	(n = 129)		(n = 39)	(n = 33)	(n = 72)	
**Pretransplant variables**								
Median age (range), years	48.5(18–69)	52(19–69)	50(18–69)	**0.03**	1(0.17–17)	2(0.08–17)	1(0.08–17)	0.82
Gender, female/male	31/35	35/28	66/63	0.38	24/15	13/20	37/35	0.10
Obesity, n (%)	20(30.3%)	14(22.2%)	34(26.4%)	0.32	2(5.1%)	0	2(2.8%)	0.50
Underlying liver disease, n (%)								
Biliary atresia	3(4.6%)	7(11.1%)	10(7.8%)	0.20	31(79.5%)	22(66.7%)	53(73.6%)	0.29
HCC	19(28.9%)	17(27.0%)	36(27.9%)	0.85	0	0	0	-
Primary biliary cirrhosis	7(10.6%)	10(15.9%)	17(13.2%)	0.44	0	0	0	-
Hepatitis C	8(12.1%)	7(11.1%)	15(11.6%)	1.00	0	0	0	-
Fulminant hepatic failure	7(10.6%)	4(6.4%)	11(8.5%)	0.53	2(5.1%)	3(9.1%)	5(6.9%)	0.66
Primary sclerosing cholangitis	5(7.6%)	3(4.8%)	8(6.2%)	0.88	2(5.1%)	1(3.0%)	3(4.2%)	1.00
Metabolic liver disease	6(9.1%)	1(1.6%)	7(5.4%)	0.12	3(7.7%)	4(12.1%)	7(9.7%)	0.70
Neoplastic liver disease other than HCC	2(3.0%)	1(1.6%)	3(2.3%)	1.00	1(2.6%)	3(9.1%)	4(5.6%)	0.33
Hepatitis B	3(4.6%)	1(1.6%)	4(3.1%)	0.62	0	0	0	-
Other	6(9.1%)	12(19.1%)	18(14.0%)	-	0	0	0	-
Dialysis, n (%)	3(4.6%)	28(44.4%)	31(24.0%)	**<0.01**	0	4(12.1%)	4(5.6%)	**0.04**
Ascites, n (%)	42(63.6%)	50(79.4%)	92(71.3%)	0.05	25(64.1%)	17(51.5%)	42(58.3%)	0.34
Previous Roux-en-Y biliary reconstruction, n (%)	5(7.6%)	5(7.9%)	10(7.8%)	1.00	34(87.2%)	21(63.6%)	55(76.4%)	**0.03**
ABO incompatibility, n (%)	12(18.2%)	21(33.3%)	33(25.6%)	0.07	4(10.3%)	4(12.1%)	8(11.1%)	1.00
Serum albumin concentration (mean ± SD), g/dL	3.1±0.7	3.1±0.5	3.1±0.6	0.80	3.5±0.6	3.4±0.7	3.5±0.6	0.65
Serum bilirubin concentration (mean ± SD), mg/dL	11.0±11.4	8.8±10.2	9.9±10.8	0.27	11.5±8.6	9.4±10.7	10.5±9.6	0.36
Pretransplantation ICU care, n (%)	NA	4(6.4%)	NA	NA	NA	0	NA	NA
Child-Pugh score (mean ± SD)	11.3±2.6	9.5±1.9	10.4±2.4	**<0.01**	10.2±2.4	8.2±2.3	9.3±2.6	**<0.01**
MELD/PELD score (mean ± SD)	22.0±9.2	18.7±7.1	20.3±8.4	**0.02**	15.7±10.1	14.0±7.5	14.9±9.0	0.43
**Operative and post-transplant variables**								
Total operation duration (mean ± SD), min	713±159	855±200	782±194	**<0.01**	609±128	716±124	658±136	**<0.01**
Intraoperative RBC transfusion (mean ± SD), mL/kg	50.0±104	40.8±48.0	45.5±81.7	0.53	70.6±75.4	32.1±37.7	52.9±63.7	**0.01**
GRWR (mean ± SD), %	NA	0.94±0.22	NA	NA	NA	2.5±1.3	NA	NA
Segment (right), n (%)	NA	35(55.6%)	NA	NA	NA	1(3.0%)	NA	NA
Roux-en-Y biliary construction, n (%)	15(22.7%)	23(36.5%)	38(29.5%)	0.12	39(100%)	29(87.9%)	68(94.4%)	**0.04**
Repeat intraabdominal or intrathoracic surgery, n (%)	15(22.7%)	11(17.4%)	26(20.2%)	0.52	6(15.4%)	5(15.2%)	11(15.3%)	1.00
**NHSN risk index**				**0.04**				0.27
0, n (%)	7(10.6%)	2(3.2%)	9(7.0%)		6(15.4%)	7(21.2%)	13(18.1%)	
1, n (%)	34(51.5%)	23(36.5%)	57(44.2%)		30(76.9%)	20(60.6%)	50(69.4%)	
2, n (%)	23(34.9%)	36(57.1%)	59(45.7%)		3(7.7%)	6(18.2%)	9(12.5%)	
3, n (%)	2(3.0%)	2(3.2%)	4(3.1%)		0	0	0	

HCC, hepatocellular carcinoma; ICU, intensive care unit; MELD, Model for End-Stage Liver Disease; PELD, Pediatric End-Stage Liver Disease; GRWR, graft-to-recipient body weight ratio; NHSN National Healthcare Safety Network; NA, not analyzed. Bold type indicates statistically significant P values.

#### (i)Adult recipients

Among the adult recipients, the median age of 2^nd^ period group was higher than that of the 1^st^ period group (*P* = 0.03). There was no difference in gender (*P* = 0.38). The predominant underlying liver diseases were HCC (27.9%, 36 patients), primary biliary cirrhosis (13.2%, 17 patients) and chronic hepatitis C (HCV) infection (11.6%, 15 patients). None of these variables showed a significant difference between groups in each period. The mean Child-Pugh score and MELD/PELD score of the recipients in the 1^st^ period group were significantly higher than those of the recipients in the 2^nd^ period group (*P*<0.01 and *P* = 0.02).

#### (ii)Pediatric recipients

There was no difference in age and gender between the two study periods (*P* = 0.82 and 0.10). The predominant underlying liver diseases were biliary atresia (73.6%, 53 recipients), and metabolic liver disease (9.7%, 7 recipients). None of these variables showed a significant difference between groups in each period. The mean Child-Pugh score of the patients in the 1^st^ period group was significantly higher than that of the recipients in the 2^nd^ period group (*P*<0.01).

### Focus of Surgical Site Infections

#### (i) Adult recipients

The SSI rates during each study period were 30.3% (20/66) during the 1^st^ period and 41.3% (26/63) during the 2^nd^ period. Table 2 shows the focus of the SSIs. The predominant infection site was the organ/space (84.8%). There was no significant difference in the focus of the SSIs between the two periods.

**Table 2:** Site of infection after living donor liver transplantation.

Infection site	Adult recipients				Pediatric recipients			
	No. of episodes (%)			*P* value	No. of episodes (%)			*P* value
	2001–2002	2011–2012	Overall		2001–2002	2011–2012	Overall	
	(n = 20)	(n = 26)	(n = 46)		(n = 10)	(n = 10)	(n = 20)	
Superficial	2(10.0%)	1(3.9%)	3(6.5%)	0.57	0	0	0	-
Deep	2(10.0%)	2(7.7%)	4(8.7%)	1.00	3(30.0%)	0	3(15.0%)	0.47
Organ/space	16(80.0%)	23(88.5%)	39(84.8%)	0.68	7(70/0%)	10(100%)	17(85.0%)	0.47

#### (ii) Pediatric recipients

The SSI rates were 25.6% (10/39) during the 1^st^ study period and 30.3% (10/33) during the 2^nd^ study period. The predominant infection site was the organ/space (85.0%).

In each study period, the SSI rates of the adult recipients were higher than those of the pediatric recipients.

### Time of Occurrence

**Fig 1:**
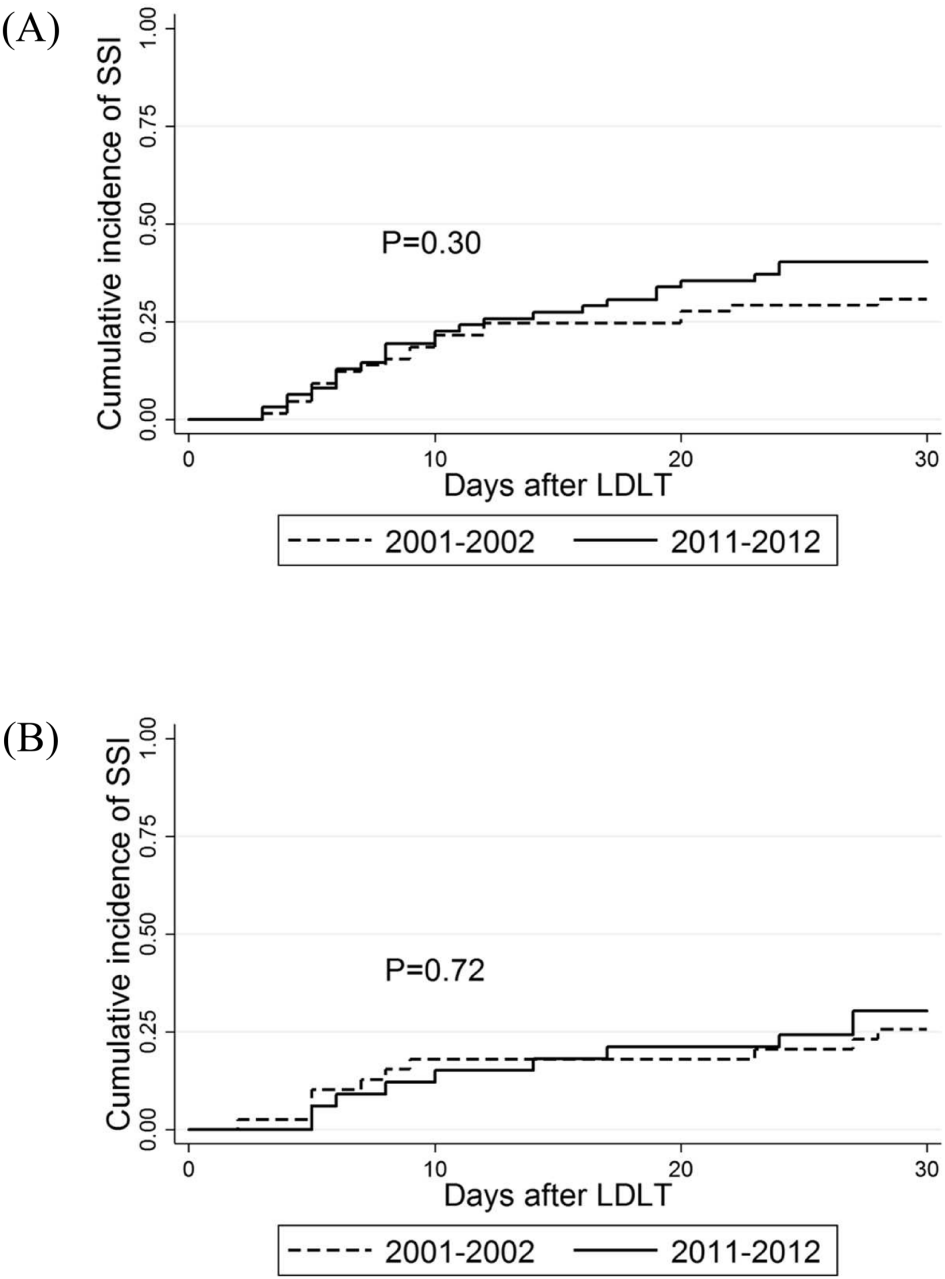
Cumulative incidence of surgical site infection after living donor liver transplantation. This figure indicates the cumulative incidence of SSIs (A: adult recipients and B: pediatric recipients). P values (determined with a Log-rank test) are indicated in each graph. SSI, surgical site infection; LDLT living donor liver transplantation.

Fig 1. shows the cumulative incidence of SSIs after LDLT. No difference was found between recipient groups in either period (*P* = 0.30 among the adult recipients and *P* = 0.72 among the pediatric recipients).

### Pathogens

Table 3 shows the causative pathogens of the SSIs.

**Table 3:** Causative pathogens of surgical site infection after living donor liver transplantation.

Pathogen	Adult recipients				Pediatric recipients			
	No. of isolates (%)			*P* value	No. of isolates (%)			*P* value
	2001–2002	2011–2012	Overall		2001–2002	2011–2012	Overall	
	(20 episodes^a^)	(26 episodes^b^)	(46 episodes)		(10 episodes)	(10 episodes^c^)	(20 episodes)	
**Gram-positive cocci**	18	12	30		5	9	14	
*Staphylococcus aureus*	10(50.0%)	3(11.5%)	13(28.26%)	**<0.01**	1(10.0%)	2(20.0%)	3(15.0%)	1.00
MRSA	10(50.0%)	2(7.7%)	12(26.1%)	**<0.01**	1(10.0%)	3(20.0%)	3(15.0%)	1.00
*Enterococcus* spp.^d^	6(30.0%)	8(30.8%)	14(30.4%)	1.00	4(40.0%)	5(50.0%)	9(45.0%)	1.00
*E*. *faecalis*	4(20.0%)	1(3.9%)	5(10.9%)	0.15	2(20.0%)	0	2(10.0%)	0.47
*E*. *faecium*	1(5.0%)	7(26.9%)	8(17.4%)	0.11	1(10.0%)	4(40.0%)	5(25.0%)	0.30
Other *Enterococcus* spp.	1(5.0%)	0	1(2.2%)	0.44	1(10.0%)	1(10.0%)	2(10.0%)	1.00
Coagulase-negative staphylococci	1(5.0%)	1(3.9%)	3(6.5%)	0.57	0	1(10.0%)	1(5.0%)	1.00
MRCNS	0	1(3.9%)	1(2.2%)	1.00	0	1(10.0%)	1(5.0%)	1.00
Other	1^e^(5.0%)	0	0		0	1^f^(10.0%)	1(5.0%)	1.00
**Gram-negative rods**	12	19	31		5	2	7	
*Pseudomonas aeruginosa*	4(20.0%)	5(19.2%)	9(19.6%)	1.00	2(20.0%)	1(10.0%)	3(15.0%)	1.00
*Enterobacter* spp.	3(15.0%)	3(11.5%)	6(13.0%)	1.00	2(20.0%)	0	2(10.0%)	0.47
*Escherichia coli*	1(5.0%)	3(11.5%)	4(8.7%)	0.62	0	1(10.0%)	1(5.0%)	1.00
*Escherichia coli* (ESBL)	0	2(7.7%)	2(4.4%)	0.50	0	0	0	-
*Klebsiella* spp.	0	2(7.7%)	2(4.4%)	0.50	1(10.0%)	0	1(5.0%)	1.00
*Klebsiella* spp. (ESBL)	0	1(3.9%)	1(2.2%)	1.00	0	0	0	-
Other	4^g^(20.0%)	5^h^(19.2%)	9(19.6%)	0.71	0	0	0	-
**Gram-positive rods**	0	2(7.7%)	2(4.4%)	0.50	0	0	0	-

SSI, surgical site infection; MRSA, methicillin-resistant *Staphylococcus aureus*; MRCNS, methicillin-resistant coagulase-negative staphylococci. Bold type indicates statistically significant P values.

^a^Including 11 episodes of polymicrobial infection.

^b^Including 4 episodes of polymicrobial infection.

^c^Including 1 episode of polymicrobial infection.

^d^Vancomycin resistance was not observed.

^e^Including a single isolate of *Streptococcus intermedius*.

^f^Including a single isolate of the *Streptococcus vestibularis*

^g^Including a single isolate of *Acinetobacter baumannii*, *Aeromonas caviae*, and *Chryseobacterium indologenes*, and 2 isolates of *Serratia marcescens*.

^h^Including a single isolate of *Acinetobacter baumannii*, *Aeromonas hydrophila*, *Sphingomonas paucimobilis*, and *Bacteroides uniformis*.

#### (i) Adult recipients

In total, 63 isolates were identified from 46 SSI patients, including 30 isolates of Gram-positive cocci (GPCs) and 31 isolates of Gram-negative rods (GNRs) and 2 isolates of Gram-positive rods. Eleven episodes during the 1^st^ period and 4 episodes during the 2^nd^ period were polymicrobial infections. All 10 isolates of *Staphylococcus aureus* (1^st^ period) and 2 of the 3 isolates of *S*. *aureus* (2^nd^ period) were resistant to methicillin. The incidence of SSIs caused by methicillin-resistant *Staphylococcus aureus* (MRSA) decreased significantly between the 2 study periods (*P*<0.01). Of the *Enterococcus* spp., *E*. *faecium* was the major causative pathogen (87.5% of enterococci) during the 2^nd^ period, whereas 1 of 6 enterococci were *E*. *faecium* during the 1^st^ period (*P* = 0.11). None of the enterococci were resistant to vancomycin. The GNRs included 9 isolates (19.6%) of *Pseudomonas aeruginosa*, 6 isolates (13.0%) of *Enterobacter* spp., 4 isolates (8.7%) of *Escherichia coli*, and 2 isolates (7.7%) of *Klebsiella* spp. Two of 3 *E*. *coli* isolates (66.7%) and 1 of 2 *Klebsiella* spp. isolates (50.0%) were extended-spectrum β-lactamase (ESBL) producers, all of which were isolated during the 2^nd^ study period.

#### (ii) Pediatric recipients

In total, 21 isolates were identified from 20 SSI patients, including 14 isolates of GPCs and 7 isolates of GNRs. Regarding causative pathogens, no significant difference was found between the two periods. One episode during the 2^nd^ period was a polymicrobial infection. Only 1 isolate (25.0%) of *E*. *faecium* was found to be a causative pathogen during the 1^st^ period, and the incidence of SSIs caused by *E*. *faecium* was increased to 80.0% (4/5 isolates) during the 2^nd^ period; in comparison, 1 of 6 enterococci during the 1st period were *E*. *faecium* (*P* = 0.30). These findings are similar to those for the adult patients. None of the enterococci were resistant to vancomycin. One isolate of ESBL-producing *E*. *coli* was found during the 2^nd^ study period.

### Risk Factors for SSIs

To determine the risk factors for SSIs, we examined 12 pretransplant variables, 6 operative and posttransplant variables, and the NHSN risk index.

#### (i) Adult recipients

A reanalysis of the previous data showed that gender (*P*<0.01) and Roux-en-Y biliary reconstruction (*P* = 0.04) were risk factors for SSIs during the 1^st^ period in the univariate analyses (Table 4). The multivariate analysis revealed that male recipients (relative risk [RR] 6.12; 95% confidence interval [CI] 1.79–20.9; *P*<0.01) and Roux-en-Y biliary reconstruction (RR 2.48; 95% CI 1.01–6.07; *P* = 0.04) were independent risk factors for SSIs after LDLT during the 1^st^ period (Table 5).

**Table 4:** Univariate analysis of risk factors for SSIs in 129 adult living donor liver transplant recipients.

Variables	2001–2002				2011–2012			
	SSIs	All others	*P* value	RR 95% CI	SSIs	All others	*P* value	RR 95% CI
	n = 20	n = 46			n = 26	n = 37		
**Pretransplant variables**								
Age, mean ± SD	42.8±15.4	44.8±11.6	0.65	0.99(0.96–1.03)	49.2±13.5	48.5±13.0	0.87	1.00 (0.97–1.03)
Gender, female/male	3/17	28/18	**<0.01**	6.16(1.80–21.1)	11/15	17/20	0.70	0.85 (0.38–1.90)
Obesity, n (%)	4(20.0%)	16(34.8%)	0.27	0.54(0.18–1.61)	8(30.8%)	6(16.2%)	0.12	1.95 (0.84–4.52)
Previous Roux-en-Y biliary reconstruction, n (%)	3(15.0%)	2(4.4%)	0.16	2.31(0.68–7.90)	3(11.5%)	2(5.4%)	0.37	1.73 (0.52–5.80)
Dialysis, n (%)	0	3(6.5%)			13(50.0%)	15(40.5%)	0.60	1.23 (0.56–2.70)
ABO incompatibility, n (%)	6(30.0%)	6(13.0%)	0.16	2.23(0.85–5.81)	13(50.0%)	8(21.6%)	**0.02**	2.59 (1.18–5.70)
Serum albumin concentration (mean ± SD), g/dL	3.2±0.72	3.0±0.68	0.55	1.22(0.65–2.29)	3.1±0.5	3.1±0.6	0.98	1.01 (0.51–2.01)
Serum bilirubin concentration (mean ± SD), mg/dL	9.9±13.7	11.5±10.4	0.19	0.99(0.94–1.03)	7.8±8.2	9.6±11.5	0.39	0.98 (0.93–1.03)
Pretransplantation ICU care, n (%)	NA	NA	NA	NA	1(3.9%)	3(8.1%)	0.51	0.51 (0.07–3.79)
Ascites, n (%)	12(60.0%)	30(65.2%)	0.72	0.85(0.35–2.08)	19(73.1%)	31(83.8%)	0.22	0.58 (0.24–1.39)
Child-Pugh score (mean ± SD), point	11.0±2.5	11.4±2.6	0.36	0.93(0.80–1.09)	9.4±1.8	9.5±1.9	0.61	0.95 (0.77–1.17)
MELD/PELD (mean ± SD), point	21.6±11.4	22.1±8.3	0.83	0.99(0.95–1.05)	17.6±6.1	19.4±7.7	0.23	0.96 (0.91–1.02)
**Operative and post-transplant variables**								
Total operation duration (mean ± SD), min	743±171	700±154	0.40	1.00(0.99–1.00)	929±231	804±161	**0.01**	1.00 (1.00–1.01)
Intraoperative RBC transfusion (mean ± SD), mL/kg	62.2±91.2	44.6±110.2	0.63	1.00(0.99–1.00)	44.5±60.1	38.2±38.2	0.75	1.00 (0.99–1.00)
GRWR (mean ± SD), %	NA	NA	NA	NA	0.88±0.20	0.99±0.23	**0.04**	7.37 (1.03–52.8)
Segment (right), n (%)	NA	NA	NA	NA	16(61.5%)	19(51.4%)	0.48	1.33 (0.60–2.97)
Roux-en-Y biliary construction, n (%)	8(40.0%)	7(15.2%)	**0.04**	2.51(1.02–6.15)	15(36.5%)	8(21.6%)	**<0.01**	2.90 (1.31–6.40)
Repeat intraabdominal or intrathoracic surgery, n (%)	7(35.0%)	8(17.4%)	0.11	2.13(0.85–5.34)	7(26.9%)	4(10.8%)	0.05	2.40 (0.99–5.75)
**NHSN risk index**			0.18	1.54(0.87–2.90)			0.74	0.90 (0.49–1.67)
0, n (%)	1(5.0%)	6(13.0%)			1(3.9%)	1(2.7%)		
1, n (%)	10(50.0%)	24(52,2%)			10(38.5%)	13(35.4%)		
2, n (%)	8(40.0%)	15(32,.6%)			14(53.9%)	22(59.5%)		
3, n (%)	1(5.0%)	1(2.2%)			1(3.9%)	1(2.7%)		

SSI, surgical site infection; RR, relative risk; MELD, Model for End-Stage Liver Disease; PELD, Pediatric End-Stage Liver Disease; RBC, red blood cell; GRWR, graft-to-recipient body weight ratio; NHSN National Healthcare Safety Network; NA, not analyzed. Bold type indicates statistically significant P values.

**Table 5:** Multivariate analysis of risk factors for SSIs in 129 adult living donor liver transplant recipients.

Variables	*P* value	RR (95% CI)
**2001–2002 (n = 66)**		
Gender (male)	<0.01	6.12 (1.79–20.9)
Roux-en-Y biliary reconstruction	**0.04**	2.48 (1.01–6.07)
**2011–2012 (n = 63)**		
ABO incompatibility, n (%)	0.16	1.83 (0.80–4.20)
GRWR (mean±SD), 1% decrement	**0.02**	7.72 (1.33–44.9)
Roux-en-Y biliary reconstruction	**<0.01**	3.18 (1.44–7.04)

SSI, surgical site infection; RR, relative risk; GRWR, graft-to-recipient body weight ratio. Bold type indicates statistically significant P values.

During the 2^nd^ period, ABO incompatibility (*P* = 0.02), longer operation duration (*P* = 0.01), lower GRWR (*P* = 0.04), and Roux-en-Y biliary reconstruction (*P*<0.01) were significantly associated with SSIs after LDLT (Table 4) in the univariate analysis. As a result of goodness-of-fit testing, we selected 3 of these 4 variables (ABO incompatibility, GRWR, and Roux-en-Y biliary reconstruction) for inclusion in the multivariate analysis. Lower GRWR (RR 7.72; 95% CI 1.33–44.9; *P* = 0.02) and Roux-en-Y biliary reconstruction (RR 3.18; 95% CI 1.44–7.04; *P*<0.01) emerged as independent risk factors for SSI after LDLT (Table 5).

#### (ii) Pediatric recipients

In the univariate analysis, age (*P* = 0.04) and repeat operation (*P* = 0.03) were significantly associated with SSI (Table 6), and repeat operation was the only independent risk factor (RR 4.75; 95% CI 1.32–17.0; *P* = 0.02) that emerged during the 1^st^ period (Table 7).

**Table 6:** Univariate analysis of risk factors for SSIs in 72 pediatric living donor liver transplant recipients.

Variables	2001–2002				2011–2012			
	SSIs	All others	*P* value	RR 95% CI	SSIs	All others	*P* value	RR 95% CI
	n = 10	n = 29			n = 10	n = 23		
**Pretransplant variables**								
Age, mean ± SD	6.2±6.8	2.8±3.9	**0.04**	1.11(1.01–1.23)	7.3±7.5	3.0±3.1	**0.01**	0.04 (1.03–1.26)
Gender, female/male	7/3	17/12	0.51	0.63(0.16–2.45)	5/5	8/15	0.50	0.65 (0.19–2.24)
Obesity, n (%)	0	2(6.9%)			0	0		
Previous Roux-en-Y biliary reconstruction, n (%)	10	24(82.8%)			8(80.0%)	13(56.5%)	0.28	2.36 (0.50–11.1)
Dialysis, n (%)	0	0			1(10.0%)	3(13.0%)	0.86	0.83 (0.10–6.55)
ABO incompatibility, n (%)	2(20.0%)	2(6.9%)	0.32	2.19(0.46–10.3)	2(20.0%)	2(8.7%)	0.37	2.02 (0.43–9.57)
Serum albumin concentration (mean ± SD), g/dL	3.7±0.5	3.4±0.6	0.10	3.16(0.80–12.6)	3.2±0.8	3.5±0.6	0.16	0.49 (0.19–1.31)
Serum bilirubin concentration (mean ± SD), mg/dL	8.8±8.8	12.4±8.5	0.23	0.95(0.88–1.03)	14.4±12.2	7.2±9.4	0.12	1.04 (0.99–1.09)
Pretransplantation ICU care, n (%)	NA	NA	NA	NA	0	0		
Ascites, n (%)	5(50.0%)	20(69.0%)	0.27	0.50(0.14–1.72)	5(50.0%)	12(52.2%)	0.91	0.93 (0.27–3.21)
Child-Pugh score (mean ± SD), point	9.5±2.3	10.4±2.5	0.28	0.87(0.68–1.12)	8.7±2.4	8.0±2.3	0.40	1.12 (0.85–1.50)
MELD/PELD (mean ± SD), point	11.9±6.7	10.7±10.9	0.17	0.94(0.87–1.02)	16.2±6.4	13.1±7.9	0.30	1.04 (0.96–1.13)
**Operative and post-transplant variables**								
Total operation duration (mean ± SD), min	639±180	598±107	0.30	1.00(0.99–1.01)	774±154	691±102	0.15	1.00 (0.99–1.00)
Intraoperative RBC transfusion (mean ± SD), mL/kg	59.4±69.1	74.4±78.3	0.36	0.99(0.99–1.01)	32.7±30.7	31.8±41.0	0.90	0.99 (0.98–1.01)
GRWR (mean ± SD)	NA	NA	NA	NA	2.1±1.3	2.6±1.2	0.23	1.42 (0.80–2.56)
Segment, right	NA	NA	NA	NA	1(10.0%)	0		
Roux-en-Y biliary construction, n (%)	10(100%)	29(100%)			9(90.0%)	20(87.0%)	0.86	1.21 (0.15–9.53)
Repeat intraabdominal or intrathoracic surgery, n (%)	4(40.0%)	2(6.9%)	**0.03**	4.75(1.33–17.0)	3(30.0%)	2(8.7%)	0.19	2.49 (0.64–9.65)
**NHSN risk index**			0.49	1.63(0.41–6.48)			**0.02**	3.38 (1.20–9.51)
0, n (%)	1(10.0%)	5(17.2%)			0	7(30.4%)		
1, n (%)	8(80.0%)	22(75.9%)			6(60.0%)	14(60.9%)		
2, n (%)	1(10.0%)	2(6.9%)			4(40.0%)	2(8.7%)		
3, n (%)	0	0			0	0		

SSI, surgical site infection; RR, relative risk; MELD, Model for End-Stage Liver Disease; PELD, Pediatric End-Stage Liver Disease; RBC, red blood cell; GRWR, graft-to-recipient body weight ratio; NHSN National Healthcare Safety Network; NA, not analyzed. Bold type indicates statistically significant P values.

**Table 7:** Multivariate analysis of risk factors for SSIs in 72 pediatric living donor liver transplant recipients.

Variables	*P* value	RR (95% CI)
**2001–2002 (n = 39)**		
Age, 1-year increment	0.09	1.09 (1.03–1.22)
Repeat intraabdominal or intrathoracic surgery, n (%)	**0.02**	4.75 (1.32–17.0)
**2011–2012 (n = 33)**		
Age, 1-year increment	**0.01**	1.14 (1.02–1.26)
NHSN risk index, 1-point increment	0.14	2.34 (0.77–7.13)

SSI, surgical site infection; RR, relative risk; NHSN National Healthcare Safety Network. Bold type indicates statistically significant P values.

During the 2^nd^ period, age (*P* = 0.01) was also a significant risk factor for SSI in the univariate analysis (Table 6); furthermore, this age was independent risk factor (RR 1.14; 95% CI 1.02–1.26; *P* = 0.01 [Table 7]).

### Prognosis

Seventeen of the adult recipients with SSI died (37.0%, 17/46 patients) in the hospital. However, overall 30-day mortalities were 10.0% (2/20 SSI patients) for the 1^st^ period group and 3.9% (1/26 SSI patients) for the 2^nd^ period group.

None of the pediatric patients with SSI died within 30 days during either of the two periods.

## Discussion

SSIs after LDLT are a major complication, with an incidence ranging from 30% to 40% [7,15]. This high incidence is similar to previously reported incidences but higher than the rates after cadaveric liver transplantation reported by Hellinger et al (16%) and Park et al (11.2%) [8,9,16]. Hellinger et al explained that the differences in the SSI incidences reported in our prior study and their study mainly resulted from case definitions and times of infection onset. They suggested that it was necessary to consider the possibility of risk factors in addition to surgical procedures for infections that occurred more than 30 days after liver transplantation. We included SSIs that had occurred more than 30 days after an LDLT in our previous study. Therefore, we defined SSIs according to the NHSN system and reanalyzed the data of the 1^st^ period. The SSI rate after LDLT seems to be higher than the rate of after cadaveric liver transplantation [8,9,16,17]. The difficulty of the surgical procedure might be one reason for this finding. The total operation duration in our study was longer than those reported in other studies of cadaveric live transplantation [9,16]. Relative to these other studies, our study also involved a higher mean intraoperative red blood cell transfusion volume [9,16]. Furthermore, the MELD/PELD scores in our study were higher than those reported in previous studies [8,18]. These factors cannot be discounted as risk factors for SSI after LDLT because they affected most of the patients examined in this study. Another reason for the higher incidence is possible over-diagnosis. The diagnosis of cholangitis is occasionally difficult in LDLT recipients because its signs, including fever, increased serum bilirubin levels and liver enzyme elevation, are similar to those of patients experiencing graft rejection. Although two infection-control doctors diagnosed cholangitis, the possibility of over-diagnosis should be considered.

Given the high incidence of SSI observed in this study, the overall 30-day mortality rates among SSI patients were extremely low. Earlier intervention by an infection control-team may improve the prognosis of SSI after LDLT. Further investigations should be conducted to assess this finding.

*S*. *aureus* and enterococci were the two major GPCs identified as causative pathogens among the LDLT recipients with SSIs. Although the incidence of SSIs caused by *E*. *faecalis* decreased, the incidence of SSIs caused by *E*. *faecium* during the 2^nd^ period increased to 26.9% compared with 5.0% during the 1^st^ period, among the adult recipients. Among the pediatric recipients, SSIs caused by *E*. *faecium* also increased, from 10.0% to 40.0%. The perioperative antibacterial prophylaxis changes that were initiated in 2003 appeared to be associated with this increased incidence of *E*. *faecium*. In a previous study of bacteremia after liver transplantation, *E*. *faecalis* was the major causative species among enterococcal bacteremia [19]. In that study, only flomoxef was used as perioperative antibacterial prophylaxis throughout the entire study period. These findings suggest that prophylaxis directed specifically against *E*. *faecalis* may decreases the incidence of SSIs caused by *E*. *faecalis* but may increase the incidence of *E*. *faecium* SSIs after LDLT.

Regarding the GNRs identified as causative pathogens of SSIs after LDLT, it is notable that ESBL producers were detected among *E*. *coli* and *Klebsiella* spp. during the 2^nd^ period. Other studies have reported infectious complications caused by ESBL-producing Enterobacteriaceae after liver transplantation [20,21]. Third-generation cephalosporins, which usually have no effect against ESBL producers, are commonly used as a perioperative prophylaxis in liver transplantation worldwide [19,22]. Therefore, ESBL producers could become a serious problem in the near future from the standpoint of the prevention and control of SSIs after LDLT. Previous studies revealed that the unnecessary use of antimicrobials has resulted in the emergence and dissemination of antibiotic-resistant nosocomial pathogens and that antimicrobial prophylaxis after wound closure was typically unnecessary [23]. The prolonged use of prophylactic antimicrobials for perioperative prophylaxis is among the major unnecessary uses of antibiotics. The Surgical Infection Prevention Project recommended that prophylactic antimicrobials be discontinued within 24 hr after the end of surgery [24]. Therefore, 72 hr of perioperative antimicrobial use could contribute to an increased incidence of drug-resistant pathogens, such as ESBL-producing Enterobacteriaceae and *E*. *faecium*. Our current antimicrobial prophylactic strategy, especially the duration of prophylactic antimicrobial use, should be considered an improvement.

Previous studies have reported that the risk factors for SSIs after liver transplantation include choledochojejunal or hepaticojejunal reconstruction, reoperation, prolonged intraoperative time, posttransplant renal replacement therapy, and a high pretransplantation model for the end-stage liver disease score [17,25,26]. In this study, Roux-en-Y biliary reconstruction was an independent risk factor for SSIs after LDLT among the adult recipients in both periods. Other independent risk factors, including lower GRWR among the adults recipients, age, repeat surgery among the pediatric recipients were also similar to the previous report [17,26]. Although we identified some significant risk factors for SSIs after LDLT, these variables appeared to be unmodifiable. Unfortunately, the change in antimicrobial prophylaxis from flomoxef to ampicillin and cefotaxime did not appear to contribute to a reduction of SSIs or an improvement of the prognosis. There were no differences in the incidence of SSIs after LDLT between the two periods. Further studies are needed to determine modifiable risk factors and an effective prevention strategy for reducing the incidence and improving the outcome of SSIs after LDLT. Furthermore, consideration of improved SSI management and prophylactic strategies is important for reducing the mortality of SSIs. Earlier antimicrobial intervention based on the results of surveillance cultures might play a key role in preventing and managing SSIs [27].

Certain limitations of this study should be noted. First, although, we conducted the multivariate analysis to determine the risk factors for SSIs in each study group, a small number of SSIs could have caused instability in the multivariable logistic regression model. Second, this study was conducted at a single center. The emergence of drug-resistant pathogens may be affected by the rate of resistant pathogens at our institute or in this area. Our institute is located in the Kyoto-Shiga region, and in this area, the rate of ESBL-producing *E*. *coli* has increased obviously in recent years [28].

In conclusion, the incidence of SSIs after LDLT remains very high. The incidence of *E*. *faecium* SSIs has increased noticeably, and we should pay attention to ESBL-producing Enterobacteriaceae as causative pathogens of SSIs after LDLT. This study revealed that Roux-en-Y biliary reconstruction was an independent risk factor for SSIs after LDLT in the adult patients. Lower GRWR was also an independent risk factor for SSI in the adults. These risk factors were similar to those reported in previous studies. To improve the SSI rate and the mortality of SSIs, changing only the prophylactic antimicrobials is not sufficient. The creation of a stricter or more individualized strategy for preventing and managing SSIs, including via prophylaxis and management, is urgently needed.
